# Experimental research on intraocular aqueous flow by PIV method

**DOI:** 10.1186/1475-925X-12-108

**Published:** 2013-10-21

**Authors:** Hongyu Yang, Hongfang Song, Xi Mei, Lin Li, Xineng Fu, Mindi Zhang, Zhicheng Liu

**Affiliations:** 1 School of Biomedical Engineering, Capital Medical University, Beijing 100069, China; 2 School of Mechanical and Vehicular Engineering, Beijing Institute of Technology, Beijing 100081, China

**Keywords:** Aqueous humor flow, PIV, Flow field, Velocity vector

## Abstract

**Background:**

Aqueous humor flows regularly from posterior chamber to anterior chamber, and this flow much involves intraocular pressure, the eye tissue nutrition and metabolism.

**Purpose:**

To visualize and measure the intraocular flow regular pattern of aqueous humor.

**Method:**

Intraocular flow in the vitro eyeball is driven to simulate the physiological aqueous humor flow, and the flow field is measured by Particle Image Velocimetry(PIV). Fluorescent particle solution of a certain concentration was infused into the root of Posterior Chamber(PC) of vitro rabbit eye to simulate the generation of aqueous and was drained out at a certain hydrostatic pressure from the angle of Anterior Chamber(AC) to represent the drainage of aqueous. PIV method was used to record and calculate the flow on the midsagittal plane of the eyeball.

**Results:**

Velocity vector distribution in AC has been obtained, and the distribution shows symmetry feature to some extent. Fluorescent particle solution first fills the PC as it is continuously infused, then surges into AC through the pupil, flows upwards toward the central cornea, reflecting and scattering, and eventually converges along the inner cornea surface towards the outflow points at the periphery of the eyeball. Velocity values around the pupillary margin are within the range of 0.008-0.012 m/s, which are close to theoretical values of 0.0133 m/s, under the driving rate of 100 μl/min.

**Conclusions:**

Flow field of aqueous humor can be measured by PIV method, which makes it possible to study the aqueous humor dynamics by experimental method. Our study provides a basis for experimental research on aqueous humor flow; further, it possibly helps to diagnose and treat eye diseases as shear force damage of ocular tissues and destructions on corneal endothelial cells from the point of intraocular flow field.

## Background

Aqueous humor, the clear, colorless intraocular flowing fluid, is secreted by ciliary and first fills the PC, then flows into the AC through the pupil, and is finally absorbed through trabecular meshwork at the angle of AC. This flow process plays an important role in intraocular pressure, the eye tissue nutrition and metabolism [1,2]. The physiological flow of aqueous humor is affected and regulated by multiple factors, and any pathological change can possibly interfere with its normal flow, then increased intraocular pressure, corneal endothelial damage, lens forward, or iris degeneration will arise [3,4].

Two methods are currently used for such flow process: computer-based theoretical calculation and measurement of the flow by experimental method. Although numerical simulation is one of the major tools for aqueous humor flow research [5-7], it is quite difficult to perform because of the complexity of the 3-dimensional anatomical structure of AC. Alternatively, fluid flow in the AC can be visualized and measured by using various particles in the fluid. Experimental methods to trace fluid flows as traditional tracer methods, considering the necessity of trace markers as fluorescent nanoparticles [8], quantum dots probes [9], or fluorescent microspheres [10] to combine with proteins which are barely contained in aqueous humor, are not well prepared to service the aqueous humor flow measurement.

In our study, PIV is applied to intraocular flow field measurement. As a whole flow field measurement system, PIV can be intuitive and non-interfering to the flow field of target observation area [11,12]. It is a relatively new attempt to introduce PIV to intraocular aqueous humor flow study [13]. This method enables visualization of the direction and calculation of the velocity of fluid flow by analyzing pairs of images of small particles captured at short intervals. Using this method, we have analyzed the flow distribution in AC during the driven flow process in the vitro eyeball. Considering the low rate of aqueous generation and the importance of flow on sagittal planes of AC, we aim to achieve slower intraocular flow field visualization on sagittal planes of eyeball with PIV method.

## Methods

### Animals and vitro eyeball

We choose purebred New Zealand White rabbits of 2.5 kg weight as eye donors, provided by the Experimental Animal Center of Capital Medical University. Implement excessive anesthesia death on rabbits by rapid injection of urethane into the ear vein. Then bluntly dissect the bulbar conjunctiva, cut the rectus, and finally cut together the central retinal artery, vein and the optic nerve bundle after ligation to isolate the eyeball. Save the vitro eyeball temporarily in saline solution at room temperature. All the animal experiments reported in our study were carried out in accordance with the National Institute of Health Guide for the Care and Use of Laboratory Animals and approved by the Institutional Animal Care and Use Committee of China.

### Particle image velocimetry technology

#### Tracer particles

Polystyrene particles with 10 um diameter are selected as tracer particles in our study. They can be uniformly dispersed with a similar density to water and show a good following feature in intraocular fluid [14-16].

#### Experimental apparatus

In our study, the pivotal device are particle image velocimetry system, including Laser, CCD camera, and Synchronization and the micro-injection pump (Nd:YAG Pulse laser with single pulse energy up to 200 mJ; CCD camera, 1600 × 1200 pixels,with Sampling Frequency of 30 fps; HARVARD PHD 2000 Syringe pump); Softwares are Dynamic Studio developed by Denmark DANTEC Company and Tecplot visualization; main materials include the fluorescent particle solution of 10um particle diameter, the no-leaking vitro rabbit eyeball, 20G × 29 mm intravenous catheter, 20 ml syringe, slender and transparent tube, glass tank, and saline.

#### Velocity measurement principle of PIV

Flow situations are captured and recorded by PIV with proper parameters of CCD camera, laser intensity (0~200 mJ) and pulse frequency (0~10000 us). Finally the corresponding velocity distributions are calculated from successive particle images.

**Figure 1:**
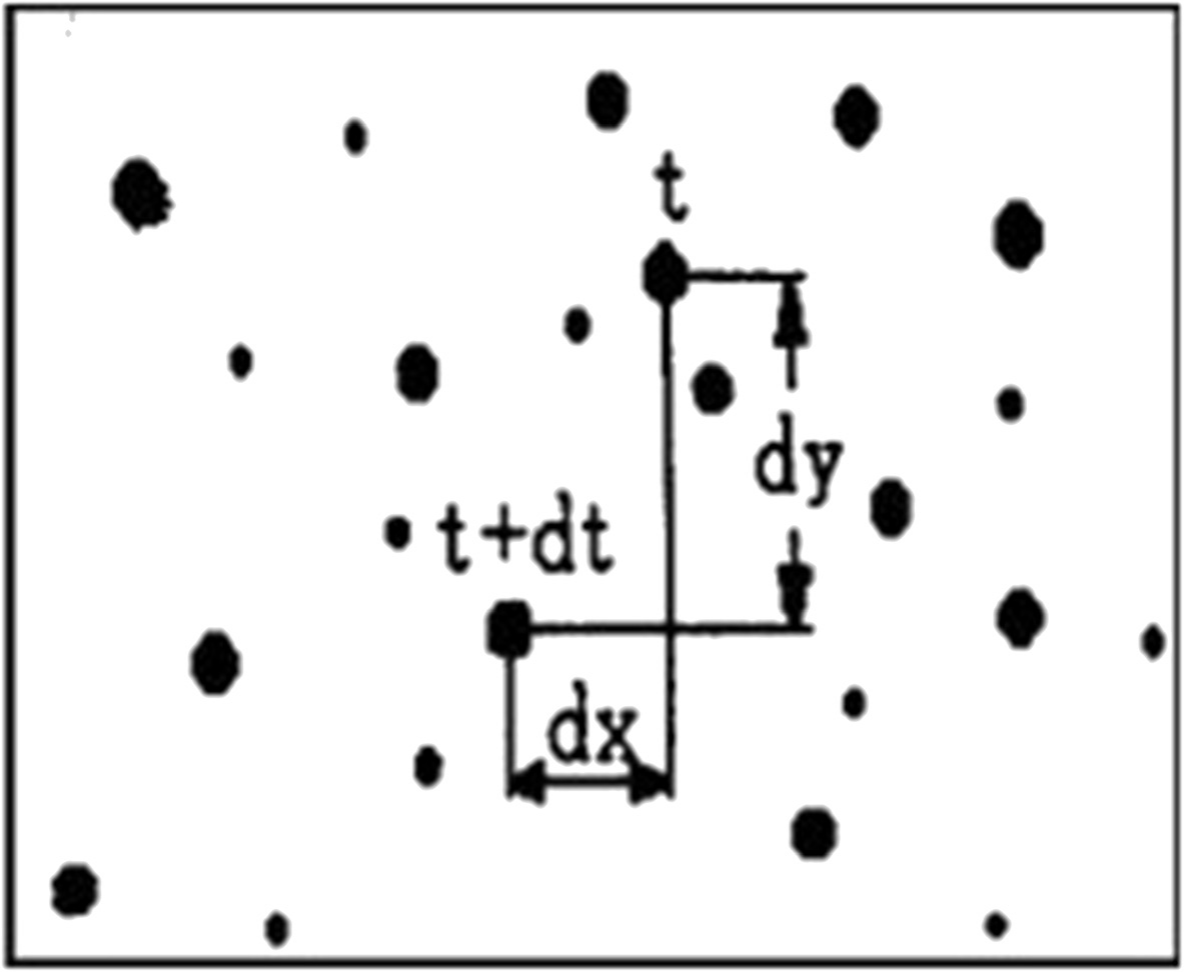
Principle of PIV velocity measurement.

As shown in Figure 1, the average velocity components of the tracer particle during a period along the x and y directions are as follows [17,18]:

V_x=dx/dt;V_y=dy/dt

Where, dt is a time period, dx and dy are the displacements of the particle along the x and y directions respectively.

In the formula above, when dt is sufficiently small, V_x and V_y can accurately reflect the instantaneous velocities of the fluid particle. PIV technology realizes the measurement of the two-dimensional flow field by measuring the instantaneous average velocity of tracer particles.

### Flow field measurement with PIV method of intraocular aqueous flow *in vitro* eyeball

#### Drive mode of aqueous humor production *in vitro* eyeball

**Figure 2:**
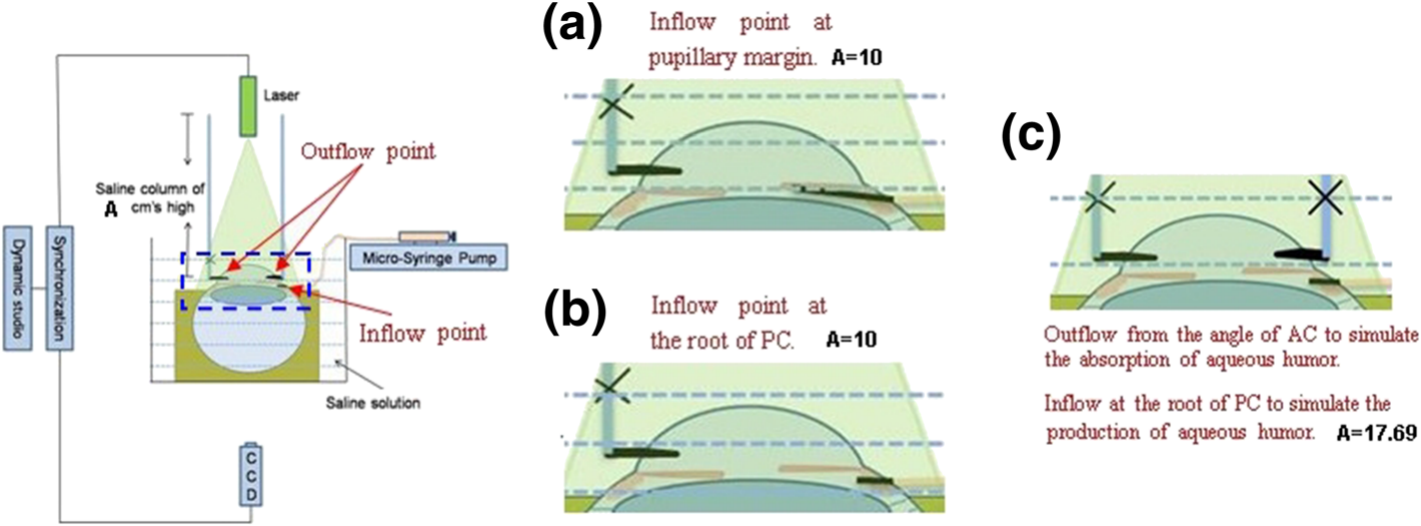
**Connection diagram of experimental apparatus. (a)** inflow point along the pupillary margin; **(b)** inflow point at the root of PC; **(c)** intraocular flow measurement in symmetry AC.

A proper inflow point in PC should be determined to better simulate the production of aqueous humor. Two typical inflow positions in PC were considered: along the pupillary margin; at the root of PC. Figure 2 is the scheme of these two conditions, in the left diagram, inflow point is along the pupillary margin; in the right diagram, inflow point is at the root of PC [13,19].

As shown in Figure 2 (a), intravenous catheter was needled at the limbus peripheral 1 mm obliquely upward in the direction of 15°, and was slided underbelow the iris in the direction of the pupil center until its tip reached the pupil edge. Then pull out the metal needle, and fix well the trocar, ensuring that the inflow point is along the pupillary margin. In the circumferential direction of 180° to the inflow point, puncture at the angle of AC as the outflow pathway for intraocular liquid, exerting a 10 cm high saline water column pressure to represent the episcleral venous pressure resistance on physiological humor outflow. This entire device was then fixed into saline to eliminate the refractive error in the measurement. Fluorescent particle solution was pumped uniformly to the pupillary margin to drive the intraocular flow by Micro-injection pump and the flow process was captured and recorded by PIV.

In Figure 2 (b), intravenous catheter was needled at the limbus peripheral 1 mm, penetrating the sclera, and was slided underbelow the iris. Pull out the metal needle for 2 mm, and continually slide the trocar until its tip reaches the root of iris. Extract the whole metal needle and fix well the trocar to ensure that the injection point is at the root of PC. Puncture at one point of angle of AC as the outflow pathway for intraocular fluid, exerting a 10 cm high saline water column pressure to represent the episcleral venous pressure resistance on physiological humor outflow. This entire device was fixed into saline to eliminate the refractive error in the measurement. Fluorescent particle solution was uniformly pumped to the root of PC to drive the intraocular flow with Micro-injection pump and this flow process was measured by PIV.

By comparing the intraocular flow fields obtained from these two conditions, proper inflow point was selected to drive the intraocular flow complying with physiological laws. In our study, inflow at the root of PC can better simulate the generation of aqueous humor.

#### Measurement of flow field in AC by PIV

Considering the symmetry of AC, we have decided to puncture two outflow points at the angle of AC, which are circumferentially 180°apart on one sagittal plane of the eyeball, to simulate the aqueous absorption process. Figure 2 (c) is the scheme of the experimental apparatus in this experimental condition.

As shown in Figure 2 (c), fluorescent particle solution was infused to the root of PC acting as the generation of aqueous. Two outflow points were punctured at the angle of AC, which are circumferentially 180° apart on one sagittal plane of the eyeball, to simulate the aqueous outflow on one sagittal plane. For the vitro eyeball with two outflow points in its AC, the AC is easier to collapse because of the instantaneously rapid outflow of intraocular liquid. And to prevent this phenomenon, we have elevated the saline column height to 17.69 cm to represent the episcleral venous pressure resistance on aqueous outflow. This entire device was fixed into saline to eliminate the refractive error in the measurement. Fluorescent particle solution was uniformly pumped to the root of PC to drive the intraocular flow with Micro-injection pump and the flow would be recorded and calculated with PIV.

## Results

### Intraocular flow fields induced by different positions of inflow points in PC

**Figure 3:**
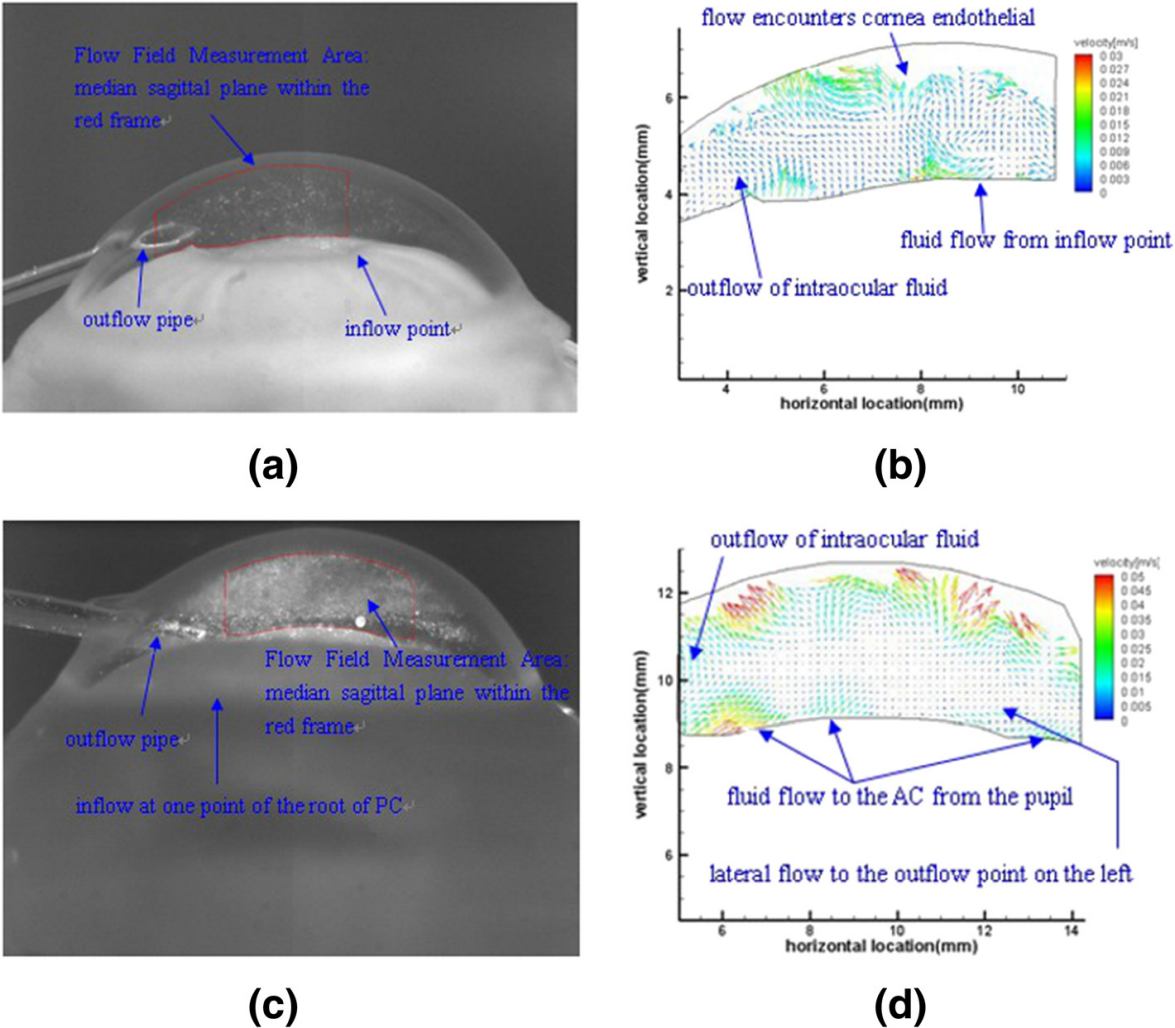
**Influence of inflow points on intraocular flow field.** Inflow point at pupillary margin in **(a)** and **(b)**; inflow point at the root of PC in **(c)** and **(d)**.

Different infusing positions in PC lead to distinct distributions of intraocular flow fields, and Figure 3 displays the results of the two typical conditions at the pump rate of 200 μl/min.

As shown in Figure 3 (a) and (b), the infusing point is along pupillary margin. Micro-injection pump uniformly pumps fluorescent particle solution to the edge of pupil at the rate of 200 μl/min. This flow rate gushes out of the tube with an inner diameter of 0.62 mm, and the theoretical value of the pouring rate is 0.011 m/s. From Figure 3 (b), the experimental vector distribution, we obtain the velocity values around the infusing point are within the range of 0.009~0.018 m/s, which are close to theoretical value. The flow pattern under this condition can be described as follows: When the influent point is along pupillary margin, the arising flow tends to go directly upwards through the pupil and into the AC, then flows straight to the outflow point at one side of the angle of AC.

Intraocular flow driven by injection at the root of PC is shown in Figure 3 (c) and (d). Micro-injection pump uniformly pumps fluorescent particle solution to the root of PC at the rate of 200 μl/min. This flow rate fills a 0.06 ml volume of PC and then overflows uniformly along the pupillary margin, through a surface with a 12.6 mm circumference and a 10 um height. The theoretical value of the overflow rate along the pupillary margin is 0.0265 m/s. From Figure 3 (d), the experimental vector distribution, we obtain the velocity values along the pupillary margin are within the range of 0.01~0.025 m/s, which are a bit smaller than theoretical value, and this phenomenon may derive from the frictional resistance of the inferior surface of the iris. The experimentally observed flow law under this condition can be described as follows: When the infusing point is at the root of PC, fluorescent particle solution first fills the PC, surges into the AC from the pupil, flows upwards toward the central cornea, reflecting and scattering, and eventually converges along the inner cornea surface towards the outflow point at the periphery of the eyeball, which complies with the physiological flow law of aqueous humor.

### Flow field in AC

**Figure 4:**
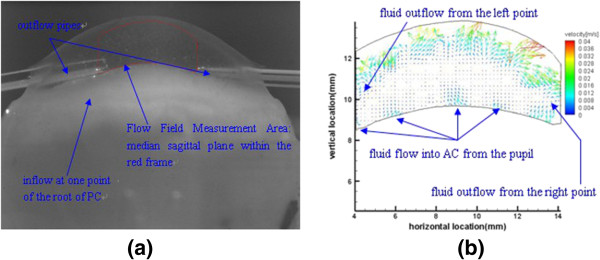
**Flow field on the midsagittal plane of AC. (a)** particle image captured by CCD; **(b)** velocity distribution of flow.

As shown in Figure 4 (a), we punctured two outflow points, which are circumferentially 180°apart on one sagittal plane of the eyeball, at the angle of AC to simulate the aqueous absorption process in the symmetry AC. Micro-injection pump uniformly pumps fluorescent particle solution to the root of PC at the rate of 100 μl/min. And the according vector distribution is in Figure 4 (b).

When the flow rate of 100 μl/min fills a 0.06 ml volume of PC and then overflows uniformly along the pupillary margin, through the surface with a 0.0126 m circumference and a 10 um height, the theoretical value of the overflow rate along the pupillary margin is 0.0133 m/s. From the vector distribution figure, we can see the left-right symmetry feature of vector distribution overall. Fluorescent particles surge upward towards the central cornea from the pupil area with the rate of 0.008~0.012 m/s, reflecting and scattering on the inner surface of cornea as converge along the cornea to outflow points at periphery of the eyeball.

## Discussion

Numerical simulation is one of the major tools for aqueous humor flow research, it is quite difficult to perform because of the complexity of the 3-dimensional anatomical structure of AC. Computer simulation of flow field in eye was investigated by Villamarin et al. [7] with some simplifications in anatomical structure, and velocity values were calculated using the boundary condition of aqueous production rate of 3 μl/min. The flow pattern in our study is similar to computer simulation, although velocity values are different to some extent. In our study, driving rate of 100 μl/min was applied to produce an intraocular flow rate range of 0.008~0.012 m/s; In calculation of our study, when driving rate was set to 3 μl/min, the theoretical value of flow rate around pupil margin was 0.398 mm/s. According to Similarity Principle in hydromechanics, when driving rate was about 30 times lower, velocity value of 0.398 mm/s is approximately 30 times lower than 0.008~0.012 m/s.

Alternatively, fluid flow in the AC can be visualized and measured by using various particles in the fluid. In our research, we try to drive the aqueous humor flow in the vitro eyeball by infusing liquid to the root of PC as the generation of aqueous and puncturing at the angle of AC with a certain pressure as the absorption of aqueous. With PIV method, we have captured and recorded the entire flow process and have calculated the corresponding velocity distributions. Visualization of this entire flow process with both 'aqueous humor secretion’ and 'aqueous humor absorption’ helps to deepen our understanding of intraocular aqueous humor flow.

Although our study promotes a method to investigate flow in eyes and has obtained much valuable experimental data, there are several limitations to be improved. The entrance of the outflow pipe is too close to the pupil, which is much different from the real absorption location of aqueous humor; so the outflow pathway needs to be redesigned to make its entrance closer to the angle of AC. There are only two symmetry outflow points in AC in our study to simulate the absorption of aqueous humor. Considering the mutual interfere of intraocular flows, they are not sufficient. Although, relatively, the number of infusing points has little effect on the pattern of intraocular flow field. The more outflow points are along the angle of AC, the closer to the real aqueous humor absorption situation. In addition, flow field of aqueous humor is also much affected by temperature gradient and gravity effect in AC, and these two factors will be considered in future.

## Conclusion

Our study provides a basis for experimental research on aqueous humor flow. With PIV method, we have captured and recorded the entire flow process and have calculated the corresponding velocity distributions; we found that aqueous humor urges into the AC from the pupil, flows upwards toward the central cornea, reflecting and scattering, and eventually converges along the inner cornea surface towards the outflow point at the periphery of the eyeball, this phenomenon complies with the physiological flow law of aqueous humor.

## Abbreviations

AC: Anterior chamber; PC: Posterior chamber; PIV: Particle image velocimetry.

## Competing interests

The authors declare that they have no competing interests.

## Authors’ information

Co-author: Hongyu Yang and Hongfang Song.
